# Long-Term Effects of Activity Status in the Elderly on Cardiorespiratory Capacity, Blood Pressure, Blood Lipids, and Body Composition: A Five-Year Follow-Up Study

**DOI:** 10.1100/tsw.2003.66

**Published:** 2003-08-20

**Authors:** Eli Carmeli, Pini Orbach, David T. Lowenthal, Joav Merrick, Raymond Coleman

**Affiliations:** Department of Physical Therapy, Sackler Faculty of Medicine, Tel Aviv University, IL-69978 Ramat Aviv, Israel

**Keywords:** elderly, aging, functional ability, physical training, activity status, body composition

## Abstract

It is generally recognized that physical activity levels in the elderly do not remain constant over time, and typically there is a marked reduction in physical activities in the elderly. The long-term benefits of regular physical training programs in the elderly are still not fully understood. This is a study of 55 elderly healthy subjects (over 65 years old) and re-evaluated for the effects of different physical activity patterns (sedentary, moderately active, and highly active) on several physiological parameters (pre- and post-training) after a 5-year period (5.30 ± 1.14 years). Measurements included: body composition, blood lipid profiles, resting systolic and diastolic blood pressure, maximal oxygen uptake, and pulmonary function. Results indicated a larger decrease in maximal oxygen uptake (VO_2max_) in the group of elderly sedentary individuals (1.5 ± 0.5 l/min) compared to the moderately active (1.7 ± 0.6 l/min) and the highly active groups (1.9 ± 0.4 l/min). An active lifestyle was not sufficient to increase the physiological function of an individual.This study could not clearly demonstrate favorable differences for the physically active groups over the sedentary group with regard to several important physiological factors over the 5-year follow-up and it appears that the recommendation for, and the initiation of, adopting active lifestyles may not be sufficient on their own to significantly increase an individual's physiological functioning.

## INTRODUCTION

By the year 2030, it is estimated that the number of individuals aged 65 years or older will reach 70 million in the U.S. alone. Moreover, people aged 85 years or older will be the fastest growing segment of the population[1]. Overall deterioration in physical activity and physiological functioning are commonly associated with aging in the elderly. The rate of aging is widely variable among individuals, and specific body systems within individuals may show a variable age-associated functional decline[2,3]. There is some evidence that appropriate exercise training in healthy elderly persons may help maintain functional capacity and quality of life[4,5]. The benefits of regular physical activity in improving health-related fitness and reducing the risk factors for coronary heart disease (CHD) have received considerable attention, especially in middle-aged males[6]. The Heritage Family Study is the most recent study that addressed the question of individual variation in responsiveness to regular exercise training[7]. Regularly performed endurance exercise training may partially protect against the increase in body fat content with aging[8]. Men and women undertaking a high level of endurance exercise training with advancing age appear to avoid many of the undesirable changes in body composition, muscle strength, and fat distribution that typically occur with aging[9]. Adult men, who performed aerobic training for 20 weeks, showed significant improvements in $VO_{2max}$, maximal expired ventilation (VEmax), and resting heart rate (RHR)[10].

Other studies have also shown that active lifestyles can have beneficial effects with regard to CHD risk factors, not only in young adults, but also in middle-aged and older adults[10,11]. Furthermore, the benefits of exercise in reducing CHD risk factors, such as elevated blood pressure (BP) and elevated cholesterol levels, while increasing muscle strength[13], body weight (BW), working capacity, and respiration ($VO_{2max}$ and VEmax) have been demonstrated[14,15,16]. Despite increasing evidence indicating the potential beneficial effects of regular physical activity on health and well being in aging adults, there has been relatively little improvement in reducing sedentary behavioral patterns among aging adult Americans during the past 20 years.

Most of the current knowledge regarding the influence of modifying physical activity levels on related physiological functions and aerobic fitness has been derived from longitudinal studies of middle-aged and elderly individuals[17]. In these studies, individual well-established physical activities patterns and exercise training protocols were relatively short or medium term (4–6 months) and make it difficult to draw conclusions regarding the long-term impact of changing physical activity status on cardiovascular functional capacity in the elderly. It is generally recognized that physical activity levels in adults and the elderly do not remain constant over time, and typically there is a marked reduction in physical activities in the elderly. The long-term benefits of regular physical training programs in the elderly are still not fully understood.

This longitudinal follow-up study in independently living men and women was designed to try and determine the impact of change or maintenance in physical activity status on body composition, cardiorespiratory fitness, and blood lipid profiles in a population of healthy people over the age of 65 years.

## METHODS

### Subjects

A group of 95 elderly subjects, aged 65 years or older from Gainesville, FL, volunteered to participate in the study. All subjects had randomly participated in exercise or control groups in prior aging studies.

These previous series of studies were designed to try to evaluate the effects of exercise training on elderly individuals. In these earlier studies, aerobic training consisted of three sessions per week for 26 weeks. Training involved walking at 50% $VO_{2max}$ with intensity gradually increasing until subjects could walk at 60–85% of their $VO_{2max}$[18,19,20]. All subjects had mainly nonactive behavior patterns prior to their initial participation (T1) in the training programs. Telephone screening was used to identify suitable healthy subjects for the exercise testing program and to determine that they did not suffer from cardiac disorders, peripheral vascular disease, pulmonary disease, or orthopedic limitations. On an initial visit, all participants in the study provided signed informed consent, and completed questionnaires regarding their overall activity status, demographics, and medical history. Subjects meeting the requirements were scheduled for a further screening visit. All procedures were approved by the College of Medicine Institutional Review Board, University of Florida.

### Procedures

In this longitudinal study, all participants were evaluated prior to and following the 5-year study period. Three sets of time tables were defined: T1, initial evaluation; T2, at the end of a training program; and T3, 5 years after the completion of the training program. All testing procedures and instruments were identical at T1, T2, and T3.

#### *Assessment of Life Quality*

The general health-related quality of life was measured at T1, T2, and T3. Subjects completed the SF-36 health questionnaire which includes eight physical and mental health scales of a total of 36 items[21], and in addition the Stanford Usual Physical Activity Questionnaire was completed by each participant. Correlation coefficient of this questionnaire was found the best ($r = 0.75$) compared with other validated measurements[22]. Participants were given a list of typical activities divided into four activity categories: light, moderate, hard, and very hard. Activities were classified according to energy requirements and expressed in terms of metabolic equivalents. The metabolic equivalent values were multiplied by the number of hours spent in each activity level and summed across categories to estimate total energy expenditure in terms of kcal/kg of body weight/day (kcal/kg/day). Subjects scoring 35 kcal/kg/day or less were classified as **sedentary** (SED); scores between 35–40 kcal/kg/day were classified as **moderately active** (MOD); scores exceeding 40 kcal/kg/day were classified as **highly active** (HA).

#### *Health Screening*

Participants underwent health screening in the laboratory after a minimum 3-h fast, 12-h abstinence from alcohol, decongestants, and tobacco products, and 24 h without vigorous exercise. Participants underwent a general medical examination and physical health history. Tests included a resting standard 12-lead electrocardiogram (ECG), and a modified Naughton treadmill protocol for graded exercise test (GXT)[16]. Before starting the test, supine and resting hyperventilation 12-lead ECG (Quinton Instrument Co., Seattle, WA; model Q2000), resting systolic blood pressure (RSBP), and resting diastolic blood pressure (RDBP) were recorded. Standard criteria for exclusion from maximal GXT were used[1]. This test was considered a diagnostic measure to determine patient suitability to continue further testing. The test was terminated if the subject was unable to continue or when symptoms of cardiovascular decompensation became evident. Both a 12-lead ECG and HR were monitored continuously throughout the exercise and during 7 min of recovery. The reliable testing[23] instruments and protocol kept constant over all testing periods. Diastolic blood pressure (DBP) and systolic blood pressure (SBP) were monitored pre-exercise, during exercise, and during the recovery period. In addition, perceived exertion (RPE) was determined at the end of each minute during exercise and at the termination of exercise[24]. All 95 subjects were scheduled for the GXT screening; 36 were excluded from the study due to medical reasons and 4 dropped out of the study.

### Measurements and Study Design

The following measurements were taken from all subjects in four separate consecutive visits with a minimum of at least two full days rest between visits: (first visit) height, body weight, skinfold thickness, $VO_{2max}$; (second visit) RHR and BP, blood chemistry, pulmonary function test, $VO_{2max}$; (third visit) RHR and BP, $VO_{2max}$; (fourth visit) physical performance test and $VO_{2max}$. Thus, $VO_{2max}$ was longitudinally assessed among the three activity groups.

### Blood Chemistry

Subjects reported to the laboratory after a minimum 12-h fast. After 15 min sitting quietly, resting BP and HR were measured. Then, a 15-ml sample of venous blood (medial cubital vein) was taken for routine screening of blood cell count (BCC) and biochemistry profiles. All blood analyses were performed at the GlaxoSmithKline Clinical Laboratories (Tampa, FL).

### Body Weight

Body weight was measured shoeless on a digital scale (Detecto Scale Co., Webb City, MO; model #8430) to the nearest 0.1 kg.

### Skinfold Determinations

Body composition measurements were assessed from skinfold measurements on the right side of the body using a Lange skinfold caliper (Cambridge Scientific Industries, Cambridge, MD; model #68902). The following seven skinfold sites were used to determine body composition: chest, axilla, triceps, subscapula, abdomen, supra-iliac and thigh[18]. The landmarks and techniques used for these measurements and for subsequent calculations of body composition were based on validation studies[25].

### Maximal Oxygen Uptake Test

The $VO_{2max}$ test was performed using the same protocol as described for the GXT for all subjects who performed less than 12 min in the initial GXT[16]. However, for those who exercised >12 min on their initial GXT, the initial speed was 3, rather than 2 mph. The $VO_{2max}$ test was terminated when the subject was unable to continue or when signs or symptoms of cardiovascular abnormalities or distress became evident (see GXT). Subjects were excluded from the study if the test was terminated for anything other than subjective fatigue. Expired air was collected in meteorological balloons during the last 4–5 min of the test. The gas levels for oxygen (Model S-3A) and carbon dioxide (Model CD-3A) were determined with Ametek gas analyzers (Thermox Instruments Division, Pittsburgh, PA) calibrated with the use of precision tank gases. Volumes were measured with a Collins 120 liter chain compensated gasometer (Warren E. Collins Incorporated, Boston, MA). To ensure that a true $VO_{2max}$ had been achieved, two of the following criteria had to be met: an increase in $VO_2$ <100 ml in the final minute of exercise, and HR considered to be maximal for a subject's age.

## Resting Blood Pressure

Resting blood pressure (RBP) was measured in the left arm using a sphygmomanometer. Subjects were placed in a seated resting position in a quiet room for 15 min prior to having their RBP and RHR measured. The average of three trials was used for statistical analysis.

## Pulmonary Function Test

A flow-volume test was administered with the subject in a sedentary resting position in order to measure forced vital capacity (FVC) and forced expiratory volume for one second (FEV1.0) using a pulmonary function testing system (Med Graphics, St. Paul, MN, model #1070). The flow-volume loop test involved having the subject inspire to total capacity and perform a FVC maneuver by expiring maximally followed by a forced inspiration vital capacity maneuver. The average of three flow-volume loop tests was used for statistical analysis.

## Physical Performance Test

The physical performance test (PPT) was administrated to subjects to provide a quantifiable measure of functional capacities to undertake common activities of daily living for subjects in different age and living status[26]. Each subject was rated by the examiner according to nine tasks including: writing a sentence, simulated eating, turning 360 degrees, putting on a jacket, lifting a book and putting it on a shelf, picking up a coin from the floor, a 50-ft walk test, climbing a flight of stairs for speed, and climbing stairs for endurance.

## Data Analysis

The analysis was divided into three parts: exercise training study (pretraining [T1] and post-training [T2] comparison), longitudinal studies (T1 to current [T3] and T2 to T3 comparisons), and a cross-sectional study of HA, MOD, and SED groups at T3. The data are presented as means (M) and standard deviation (SD) for all variables. All analyses used a significance level of $p < 0.05$. To evaluate the results of the post-training programs, multiple univariate one-way factorial ANOVAs with repeated measurement on the time of measurement (T1 and T2) were used. In longitudinal analyses, multiple univariate two-way 3 (activity level: HA, MOD, SED) × 2 (time of measurement: T1 and T3 testing; T2 and T3 testing) factorial ANOVAs with repeated measures on the last variable were used. Moreover, when initial significant differences among the groups at T1 and T2 were identified, multiple univariate two-way 3 × 2 ANCOVAs with repeated measures on the second variable (time of measurement) were used. In the cross-sectional analysis (at T3), multiple univariate one-way (activity level: HA, MOD, SED) factorial ANOVAs were used. Gender interaction was evaluated by repeating the above-described analyses with the addition of sex groups (female and male) in the model of interest. In the ANOVA and ANCOVA analyses from the longitudinal and cross-sectional studies, significant F ratios ($p < 0.05$) were analyzed using the Student Newman-Keuls test.

## RESULTS

No significant gender differences for dependent variables and time points were found, consequently, combined results of both female and male subjects are presented. Out of the initial sample size of the 95 participants, 36 were excluded from the study due to medical reasons and 4 subjects dropped out, leaving 55 persons in the study (males, n = 24, mean age 66.1 ± 3.7; females, n = 31, mean age 67.5 ± 4.2).

### Effectiveness of Prior Training

Analysis of the previous training data indicated that subjects responded with a significant increase in relative $VO_{2max}$ (18%), absolute $VO_{2max}$ (l/min; $AbVO_{2max}$) (10%), and VEmax (7%). In addition, RDBP (6%), RSBP (4%), RHR (5%), body fat (5%), and sum of seven skinfolds (7%) were found to be lower after training (see Table 1).

### Pretraining and Current Comparison

Based on the Stanford 7-day recall questionnaire results, subjects were divided into three activity groups (HA, MOD, SED) for the analyses of dependent variables. No significant changes were observed in the HA group. Nevertheless, there was a nonsignificant trend indicating a decrease in relative $VO_{2max}$ (9%), % BF (8%), and a significant decrease in sum of seven skinfolds (14%) for T3 measurements (see Table 2).

Results from the MOD group indicated significant changes for % BF (12%), and relative $VO_{2max}$ (9%), revealing significant lower % BF and relative $VO_{2max}$ for T3 measurement (Table 3). For the MOD group, data analysis also indicated a significantly reduced $AbVO_{2max}$ (6%) and sum of seven skinfolds (18%) measurements for T3. Finally, T3 maximal SBP (SBPmax) was found to be higher (5%) for the MOD group.

Results from the SED group revealed a significant reduction in body fat (10%) and relative $VO_{2max}$ (18%) (see Table 4). It was evident from the results that the SED group not only had lower relative $VO_{2max}$ than MOD and HA groups, but also had a greater rate of decline in their $VO_{2max}$. Results for the SED group also revealed a significant decrease in maximal HR (HRmax) (5%), $AbVO_{2max}$ (17%), FEV1.0 (11%), and sum of seven skinfolds (17%) for T3 measurements.

### Post-Training and Current Comparison

Results for the HA group indicated a significance decrease in relative $VO_{2max}$ (20%) from T2 to T3 (see Table 5). Current results for the HA group showed smaller, but significant, decreases in $AbVO_{2max}$ (17%) than in MOD and SED groups. In addition, values for pulmonary function such as FVC (9%) and FEV1.0 (10%) were found to be reduced in a similar fashion as the MOD and SED groups. Additional T3 results for the HA group showed no significance change compared to T2. However, results for HA group indicated a substantially preferable shift in values for almost every category analyzed when compared to MOD and SED groups.

**TABLE 1**
**Pretraining and Post-Training Parameters**

| Variables | n | Pretraining (T1) | Post-Training (T2) | *p* Values |
|---|---|---|---|---|
| Age (years) | 55 | 67.23 ± 4.4 | — | — |
| Weight (kg) | 55 | 71.0 ± 13.4 | 70.9 ± 14.4 | 0.832 |
| Body fat (%) | 54 | 29.5 ± 7.1 | 28.0 ± 7.4 | 0.009* |
| $V0_2$ ($ml{\cdot}kg^{-1}{\cdot}min^{-1}$) | 44 | 25.8 ± 5.4 | 29.0 ± 6.4 | 0.009* |
| $V0_2$ (l/min) | 44 | 1.9 ± 0.5 | 2.1 ± 0.6 | 0.009* |
| HRmax (beats/min) | 44 | 163 ± 12.9 | 164.8 ± 15 | 0.360 |
| SBPmax (mm Hg) | 44 | 192.6 ± 23.1 | 195.8 ± 25.6 | 0.103 |
| DBPmax (mm Hg) | 44 | 86.5 ± 12.6 | 84.3 ± 12.9 | 0.123 |
| VEmax (l/min) | 44 | 71.3 ± 23.6 | 75.6 ± 22.6 | 0.029* |
| RSBP (mm Hg) | 55 | 123.3 ± 16.3 | 119.2 ± 16 | 0.004* |
| RDBP (mm Hg) | 55 | 74.1 ± 8.2 | 69.8 ± 9.2 | 0.009* |
| RHR (beats/min) | 55 | 66.1 ± 7.6 | 63 ± 7.1 | 0.009* |
| FEV/FVC (%) | 25 | 78.5 ± 6 | 77.2 ± 5.8 | 0.063 |
| FVC (l) | 25 | 3.5 ± 0.8 | 3.5 ± 0.9 | 0.156 |
| FEV1.0 (l) | 25 | 2.9 ± 1.2 | 2.7 ± 0.6 | 0.315 |
| Skinfold (mm) | 54 | 164.9 ± 52.9 | 153.6 ± 54.7 | 0.009* |

*Note:* Abbreviations are common for all tables presented. Data presented in all tables are for both genders combined. *Significance, $p < 0.05$. Number of observations (n). $V0_2$: maximal oxygen uptake, HRmax: maximal heart rate in 1 min, SBPmax: maximal systolic blood pressure, DBPmax: maximal diastolic blood pressure, VEmax: maximal expired ventilation, RSBP: resting systolic blood pressure, RDBP: resting diastolic blood pressure, RHR: resting heart rate in 1 min, FVC: forced vital capacity, FEV1.0: forced expiratory volume in 1 s, Skinfold = sum of seven measurements.

As in the HA group, results for the MOD group showed decreases in $VO_{2max}$ (20%), together with a significant decrease in % BF (6%) from T2 to T3 (see Table 6). Current results for the MOD group showed a significant decrease in VEmax (10%), $AbVO_{2max}$ (20%), FVC (10%), FEV1.0 (8%), and sum of seven skinfolds (12%) compared to T2 data.

Compared to the MOD and HA groups, results for the SED group showed a somewhat greater decrease in $VO_{2max}$ (28%), as well as a decrease in % BF (7%) for T3 measurements (see Table 7). Results for the SED group indicate a significant decrease in current HRmax (6%), $AbVO_{2max}$ (29%), FVC (12%), FEV1.0 (8%), and sum of seven skinfolds (14%). In addition, T3 results indicate an increase in RSBP (5%), RDBP (7%), RHR (6%), and FEV/FVC (6%).

## Cross-Sectional Comparison

Mean body weight (BW) values were significantly different among groups ($p$ = 0.031). When post hoc analysis was performed, significant differences were found between the SED and MOD groups, indicating heavier BW for the SED group than that found for the MOD group (see Table 8). The mean relative $VO_{2max}$ values were borderline, but not significantly different, among groups ($p$ = 0.050). Relative $VO_{2max}$ results, although borderline, again appeared favorable for the HA and the MOD groups, when compared to the SED group. In addition, results indicated the HA group to have significantly higher FVC values ($p$ = 0.036) than MOD and SED groups.

**TABLE 2**
**Pretraining and Current Measurements for Highly Active Subjects**

| Variables | n | Pretraining (T1) | Current (T3) | *p* |
|---|---|---|---|---|
| Age (years) | 12 | 66.8 ± 3.2 | 71.9 ± 4.0 | — |
| Weight (kg) | 11 | 74.2 ± 8.1 | 73.6 ± 9.4 | 0.597 |
| Body fat (%) | 11 | 25.2 ± 4.8 | 23.3 ± 4.6 | 0.081 |
| V02 ($ml \cdot kg^{-1} \cdot min^{-1}$) | 10 | 26.7 ± 5.7 | 24.2 ± 4.8 | 0.085 |
| V02 (l/min) | 10 | 2.1 ± 0.5 | 1.9 ± 0.4 | 0.090 |
| HRmax (beats/min) | 10 | 166.2 ± 12.4 | 161.8 ± 23 | 0.393 |
| SBPmax (mm Hg) | 10 | 198.1 ± 26.3 | 198 ± 19.0 | 0.990 |
| DBPmax (mm Hg) | 10 | 93.0 ± 14.0 | 91.0 ± 10.1 | 0.541 |
| VEmax (l/min) | 10 | 73.4 ± 18.1 | 77.3 ± 19.3 | 0.346 |
| RSBP (mm Hg) | 11 | 120.7 ± 19.3 | 118.5 ± 13.3 | 0.654 |
| RDBP (mm Hg) | 11 | 74.2 ± 9.4 | 73.3 ± 6.5 | 0.754 |
| RHR (beats/min) | 11 | 60.9 ± 7.4 | 59.9 ± 8.8 | 0.454 |
| FEV/FVC (%) | 6 | 75.2 ± 4.1 | 73.7 ± 4.7 | 0.423 |
| FVC (l) | 6 | 4.2 ± 0.7 | 4.1 ± 0.7 | 0.088 |
| FEV1.0 (l) | 6 | 3.2 ± 0.6 | 3 ± 0.6 | 0.132 |
| Skinfold (mm) | 11 | 138.1 ± 31.2 | 119.7 ± 28.3 | 0.022* |
| Cholesterol (total) | 11 | 212.4 ± 27.2 | 206.9 ± 25.2 | 0.412 |
| Triglycerides | 10 | 117.0 ± 35.1 | 123.6 ± 46.2 | 0.528 |

*Significance, $p < 0.05$.

However, FEV1.0 and FEV/FVC results indicated no significant differences among groups ($p = 0.461$ and $p = 0.214$, respectively). Analysis of the sum of seven skinfold measurements detected significantly higher values for the SED group when compared to the HA and MOD groups. However, no significant differences were found between the MOD and HA group. The SED group had lower physical function as measured by the modified nine-item physical performance test (PPT) compared to the MOD and the HA groups ($p = 0.004$).

## DISCUSSION

The primary finding of the present study was that the group of more physically active elderly individuals had maintained a better physiological status, when compared to sedentary individuals (SED) in both longitudinal as well as cross-sectional comparisons. Moreover, this study also found a decrease in important physiological measurements, such as $VO_{2max}$, skinfold and % BF, in the HA (highly active) and MOD (moderately active) active categories.

Although active elderly individuals generally maintained, but not improved, $VO_{2max}$ over the years (T1 to T3 comparison), results indicated that these elderly individuals have slightly decreased aerobic capacities. The decrease in $VO_{2max}$ for MOD elderly individuals (9%), together with the trend of decline for HA elderly individuals, are in agreement with previous studies indicating that as sedentary men age their $VO_{2max}$ decreases at a rate of approximately 0.45 $ml \cdot kg^{-1} \cdot min^{-1}$ per year[27,28]. The rate of decline in $VO_{2max}$ in MOD active elderly individuals agrees with other studies showing an age-related decline averaging 5–10% per decade[29].

**TABLE 3**
**Pretraining and Current Measurements for Moderately Active Subjects**

| Variables | n | Pretraining (T1) | Current (T3) | *p* |
|---|---|---|---|---|
| Age (years) | 27 | 68.2 ± 4.3 | 73.8 ± 4.4 | — |
| Weight (kg) | 27 | 66.0 ± 11.7 | 65.3 ± 12.5 | 0.2985 |
| Body fat (%) | 26 | 28.9 ± 7.0 | 25.7 ± 5.9 | 0.0003* |
| $V0_2$ ($ml{\cdot}kg^{-1}{\cdot}min^{-1}$) | 21 | 26.8 ± 5.9 | 24.6 ± 6.9 | 0.0141* |
| $V0_2$ (l/min) | 21 | 1.8 ± 0.6 | 1.7 ± 0.6 | 0.011* |
| HRmax (beats/min) | 21 | 163 ± 13.7 | 162.4 ± 15.3 | 0.825 |
| SBPmax (mm Hg) | 21 | 193.4 ± 22.9 | 201.8 ± 22.4 | 0.039* |
| DBPmax (mm Hg) | 21 | 86.0 ± 11.1 | 89.4 ± 14.5 | 0.276 |
| VEmax (l/min) | 21 | 73.3 ± 26.5 | 70.6 ± 26.2 | 0.441 |
| RSBP (mm Hg) | 26 | 125.0 ± 16 | 128 ± 19.1 | 0.319 |
| RDBP (mm Hg) | 26 | 74.2 ± 8.2 | 75.7 ± 6.5 | 0.265 |
| RHR (beats/min) | 26 | 66.1 ± 6.3 | 66.2 ± 7.8 | 0.923 |
| FEV/FVC (%) | 8 | 80.0 ± 6.1 | 80.1 ± 3.8 | 0.811 |
| FVC (l) | 8 | 3.0 ± 0.5 | 2.8 ± 0.6 | 0.160 |
| FEV1.0 (l) | 8 | 3.1 ± 2.0 | 2.3 ± 0.5 | 0.243 |
| Skinfold (mm) | 26 | 157 ± 49.5 | 129.1 ± 40.1 | 0.0001* |
| Cholesterol (total) | 23 | 210.9 ± 40.2 | 220.2 ± 40 | 0.197 |
| Triglycerides | 22 | 133.5 ± 52.8 | 135.0 ± 49.8 | 0.896 |

*Significance, $p < 0.05$.

**TABLE 4**
**Pretraining and Current Measurements for Sedentary Subjects**

| Variables | n | Pretraining (T1) | Current (T3) | *p* |
|---|---|---|---|---|
| Age (years) | 16 | 68.2 ± 4.3 | 73.8 ± 4.4 | — |
| Weight (kg) | 16 | 75.9 ± 16.7 | 76.8 ± 18.6 | 0.539 |
| Body fat (%) | 16 | 32.8 ± 7.1 | 29.8 ± 6.8 | 0.006* |
| $V0_2$ ($ml{\cdot}kg^{-1}{\cdot}min^{-1}$) | 10 | 23.5 ± 4.1 | 19.1 ± 4.4 | 0.005* |
| $V0_2$ (l/min) | 10 | 1.8 ± 0.5 | 1.5 ± 0.5 | 0.005* |
| HRmax (beats/min) | 10 | 159.6 ± 8.8 | 151.5 ± 13.6 | 0.011* |
| SBPmax (mm Hg) | 10 | 187.5 ± 18.3 | 194.1 ± 15.4 | 0.227 |
| DBPmax (mm Hg) | 10 | 82.1 ± 11.4 | 85.8 ± 15.4 | 0.524 |
| VEmax (l/min) | 10 | 61.6 ± 24 | 64.5 ± 23.1 | 0.219 |
| RSBP (mm Hg) | 16 | 121.8 ± 16.5 | 124.4 ± 10.1 | 0.484 |
| RDBP (mm Hg) | 16 | 73.4 ± 7.8 | 74.6 ± 5.1 | 0.487 |
| RHR (beats/min) | 16 | 69.1 ± 8.3 | 69.2 ± 8.7 | 0.973 |
| FEV/FVC (%) | 9 | 78.0 ± 5.5 | 81.6 ± 7.6 | 0.160 |
| FVC (l) | 9 | 3.5 ± 0.9 | 3.1 ± 0.8 | 0.002 |
| FEV1.0 (l) | 9 | 2.8 ± 0.7 | 2.5 ± 0.6 | 0.002* |
| Skinfold (mm) | 16 | 190.3 ± 61.8 | 159.5 ± 50.5 | 0.006* |
| Cholesterol (total) | 14 | 229.2 ± 55.5 | 222.8 ± 33.2 | 0.564 |
| Triglycerides | 14 | 119.0 ± 42.3 | 138.9 ± 60.5 | 0.155 |

*Significance, $p < 0.05$.

**TABLE 5**
**Post-Training and Current Measurements for Highly Active Subjects**

| Variables | n | Post-Training (T2) | Current (T3) | *p* |
|---|---|---|---|---|
| Age (years) | 12 | 66.8 ± 3.2 | 71.9 ± 4.0 | — |
| Weight (kg) | 10 | 74.2 ± 7.9 | 74.2 ± 9.3 | 1.000 |
| Body Fat (%) | 10 | 23.0 ± 5.4 | 22.6 ± 4.2 | 0.578 |
| V02 (ml·kg$^{-1}$·min$^{-1}$) | 9 | 31.1 ± 6.8 | 25.1 ± 4.2 | 0.009* |
| V02 (l/min) | 9 | 2.4 ± 0.4 | 2.0 ± 0.3 | 0.010* |
| HRmax (beats/min) | 9 | 164.8 ± 10.6 | 163.1 ± 23.9 | 0.818 |
| SBPmax (mm Hg) | 9 | 202.9 ± 22.5 | 198.7 ± 20.0 | 0.570 |
| DBPmax (mm Hg) | 9 | 91.8 ± 11.7 | 88.9 ± 11.7 | 0.288 |
| VEmax (l/min) | 9 | 80.1 ± 16.1 | 81.6 ± 14.8 | 0.703 |
| RSBP (mm Hg) | 10 | 111.3 ± 18.2 | 116.9 ± 12.7 | 0.256 |
| RDBP (mm Hg) | 10 | 67.8 ± 10.6 | 72.3 ± 6.0 | 0.172 |
| RHR (beats/min) | 10 | 57.3 ± 6.2 | 59.3 ± 9.1 | 0.313 |
| FEV/FVC (%) | 6 | 72.7 ± 3.3 | 73.2 ± 5.6 | 0.839 |
| FVC (l) | 6 | 4.5 ± 0.7 | 4.1 ± 0.7 | 0.049* |
| FEV1.0 (l) | 6 | 3.3 ± 0.6 | 3.0 ± 0.6 | 0.006* |
| Skinfold (mm) | 10 | 124.7 ± 33.5 | 117.1 ± 28.3 | 0.114 |

*Significance, $p < 0.05$.

**TABLE 6**
**Post-Training and Current Measurements for Moderately Active Subjects**

| Variables | n | Post-Training (T2) | Current (T3) | *p* |
|---|---|---|---|---|
| Age (years) | 27 | 68.2 ± 4.3 | 73.8 ± 4.4 | — |
| Weight (kg) | 27 | 65.6 ± 12.3 | 65.3 ± 12.5 | 0.149 |
| Body fat (%) | 26 | 27.3 ± 6.9 | 25.7 ± 5.9 | 0.032* |
| $V0_2$ (ml·kg$^{-1}$·min$^{-1}$) | 21 | 30.2 ± 5.8 | 24.6 ± 6.9 | 0.000* |
| $V0_2$ (l/min) | 21 | 2.1 ± 0.6 | 1.7 ± 0.6 | 0.000* |
| HRmax (beats/min) | 21 | 164.7 ± 16.8 | 162.4 ± 15.3 | 0.307 |
| SBPmax (mm Hg) | 21 | 197.0 ± 26.2 | 201.8 ± 22.4 | 0.266 |
| DBPmax (mm Hg) | 21 | 83.1 ± 13.0 | 89.4 ± 14.5 | 0.045* |
| VEmax (l/min) | 21 | 78.4 ± 24.9 | 70.6 ± 26.2 | 0.026* |
| RSBP (mm Hg) | 26 | 122.1 ± 17.7 | 128.0 ± 19.1 | 0.069 |
| RDBP (mm Hg) | 26 | 70.1 ± 9.3 | 75.7 ± 6.5 | 0.003* |
| RHR (beats/min) | 26 | 63.8 ± 7.0 | 66.2 ± 7.8 | 0.115 |
| FEV/FVC (%) | 8 | 79.0 ± 4.4 | 80.1 ± 3.8 | 0.540 |
| FVC (l) | 8 | 3.1 ± 0.6 | 2.8 ± 0.6 | 0.014* |
| FEV1.0 (l) | 8 | 2.5 ± 0.5 | 2.3 ± 0.47 | 0.022* |
| Skinfold (mm) | 26 | 145.3 ± 48.8 | 129.1 ± 40.1 | 0.006* |

*Significance, $p < 0.05$.

**TABLE 7**
**Post-Training and Current Measurements for Sedentary Subjects**

| Variables | n | Post-Training (T2) | Current (T3) | *p* |
|---|---|---|---|---|
| Age (years) | 16 | 68.2 ± 4.3 | 73.8 ± 4.4 | — |
| Weight (kg) | 16 | 77.0 ± 18.3 | 76.8 ± 18.6 | 0.851 |
| Body fat (%) | 16 | 32.0 ± 7.8 | 29.8 ± 6.7 | 0.017* |
| $V0_2$ ($ml{\cdot}kg^{-1}{\cdot}min^{-1}$) | 9 | 26.0 ± 6.7 | 18.8 ± 4.6 | 0.001* |
| VEmax (l/min) | 9 | 2.1 ± 0.6 | 1.5 ± 0.5 | 0.000* |
| HRmax (beats/min) | 9 | 159.1 ± 10.2 | 150.7 ± 14.2 | 0.014* |
| SBPmax (mm Hg) | 9 | 192.6 ± 28.9 | 196.6± 17.1 | 0.586 |
| DBPmax (mm Hg) | 9 | 80.2 ± 15.0 | 84.9 ± 16.0 | 0.437 |
| VEmax (l/min) | 9 | 72.1 ± 26.0 | 65.9 ± 24.1 | 0.084 |
| RSBP (mm Hg) | 16 | 118.3 ± 10.2 | 124.4 ± 10.1 | 0.009* |
| RDBP (mm Hg) | 16 | 69.4 ± 8.6 | 74.6 ± 5.1 | 0.004* |
| RHR (beats/min) | 16 | 65.4 ± 7.0 | 69.2 ± 8.7 | 0.037* |
| FEV/FVC (%) | 9 | 77.2 ± 5.9 | 81.6 ± 7.6 | 0.029* |
| FVC (l) | 9 | 3.5 ± 0.8 | 3.1 ± 0.8 | 0.002* |
| FEV1.0 (l) | 9 | 2.7 ± 0.6 | 2.5 ± 0.6 | 0.016* |
| Skinfold (mm) | 16 | 184.3 ± 65 | 159.5 ± 50.5 | 0.011* |

*Significance, $p < 0.05$.

**TABLE 8**
**Cross-Sectional Comparison for Current Measurements**

| Variables | Highly Active Group | Moderately Active Group | Sedentary Group | *p* |
|---|---|---|---|---|
| Age (years) | 71.9 ± 4.0 (12) | 72.0 ± 5.4 (27) | 73.8 ± 4.4 (16) | — |
| Weight (kg) | 73.6 ± 9.4 (11) | 65.3 ± 12.5 (27) | 76.8 ± 18.6 (16) | 0.031*[2] |
| Body fat (%) | 23.3 ± 4.6 (11) | 25.7 ± 5.8 (27) | 29.8 ± 6.8 (16) | 0.018*[1] |
| $V0_2$ ($ml{\cdot}kg^{-1}{\cdot}min^{-1}$) | 24.3 ± 4.8 (10) | 24.6 ± 6.9 (21) | 19.1 ± 4.2 (10) | 0.050 |
| HRmax(beats/min) | 161.8 ± 23.0 (10) | 162.4 ± 15.3 (21) | 151.5 ± 13.6 (10) | 0.238 |
| RSBP (mm Hg) | 118.5 ± 13.3 (11) | 128.0 ± 19.1 (26) | 124.4 ± 10.1 (16) | 0.556 |
| RDBP (mm Hg) | 73.3 ± 6.5 (11) | 75.7 ± 6.5 (26) | 74.6 ± 5.1 (16) | 0.920 |
| FEV/FVC (%) | 77.4 ± 10.0 (12) | 81.0 ± 5.6 (27) | 82.0 ± 6.3 (15) | 0.214 |
| FVC (l) | 3.7 ± 0.9 (12) | 3.0 ± 0.6 (27) | 3.1 ± 0.9 (15) | 0.036 |
| FEV1.0 (l) | 2.8 ± 0.7 (12) | 2.6 ± 0.7 (27) | 2.5 ± 0.6 (15) | 0.461 |
| Skinfold (mm) | 119.7 ± 28.3 (11) | 129.1 ± 39.3 (27) | 159.5 ± 50.5 (16) | 0.027*[1] |
| TG | 121.3 ± 44.5 (11) | 131.0 ± 48.0 (26) | 151.1 ± 70.7 (16) | 0.343 |
| LDL | 137.7 ± 27.5 (11) | 144.0 ± 37.4 (26) | 136.8 ± 32.3 (16) | 0.767 |
| HDL | 45.8 ± 9.5 (11) | 55.3 ± 15.5 (26) | 51.6 ± 17.5 (16)*[3] | 0.222* |
| CHOL | 210.3 ± 26.7 (12) | 215.5 ± 59.4 (26) | 218.5 ± 34.3 (16) | 0.900 |
| PPT | 27.3 ± 1.4 (12) | 27.8 ± 1.8 (26) | 24.8 ± 4.5 (16) | 0.004 * |

* Post hoc test: SED group were found to be significantly different from HA and MOD active groups ($p < 0.05$). Post hoc test: HA group was found to be significantly different from MOD and SED groups ($p < 0.05$).

*[1] Post hoc test: HA group is significantly different from SED ($p < 0.05$).

*[2] Post hoc test: SED group significantly different from MOD group ($p < 0.05$).

*[3] Post hoc test: HA group was found to be significantly different from MOD group.

Furthermore, the greater decrease in relative $VO_{2max}$ observed for the T2 to T3 analysis is in agreement with some of the first longitudinal studies of $VO_{2max}$[30,31,32]. Dill et al.[31] reported a greater rate of $VO_{2max}$ decline in highly trained men who had not been training during the years preceding their retesting. Similarly, the rate of decline in $VO_{2max}$ observed for the SED group was only slightly different compared to the active groups and was somewhat in agreement with previous studies. Our results suggested that healthy sedentary men and women combined (see Table 4) and separated (not published), who undergo natural aging processes, demonstrate similar rates of decline in $VO_{2max}$ as their highly active counterparts with a progressive decline in $VO_{2max}$, expressed traditionally as kg/BW generally occurring with advancing age. This decline could be attributed to the age-associated increase in % BF and BW and the loss of metabolically active tissue (i.e., muscles which decrease $VO_{2max}$ when normalized for weight). Muscle mass has been reported to decrease with age (10–12% per decade), even when total body mass is maintained, probably owing to increased fat mass. Furthermore, in both sexes, a large portion of the age-associated decline in $VO_{2max}$ in nonendurance-trained individuals can be explained by the loss of muscle mass[33]. Since BW in this study was not found to change over time, it is assumed that it did not account for the decreased $VO_{2max}$ observed. Furthermore, it is possible that the activity intensity performed by the MOD group, and to a certain degree by the HA group, was insufficient to prevent the moderate decrease in $VO_{2max}$ observed in this study.

Fat tissue accumulation and a loss of fat-free mass have been reported in advancing age resulting in an increase in BW. However, recent studies showed a consistent improvement in total BW and in skinfold measurements due to different training regimens[8]. In the present study, the longitudinal analysis of total BF indicated an insignificant decrease ($p < 0.05$) in % BF (12%) for MOD active elderly individuals as well as a mild decrease in % BF of HA elderly individuals. Therefore, our data indicate that staying physically active (i.e., engaging in moderate and high activity levels) is an important lifestyle factor that may affect age-related changes in body composition. Nevertheless, it is important to note that the decrease in % BF for the HA elderly individuals was not statistically significant, although it is possible that this was due solely to the relatively small number of observations. Contrary to the notion that a decline in physical activity performed (reduced exercise-related energy expenditure) may contribute to age-associated elevations in BF, our data indicate that whole body adiposity decreases with age in the sedentary individuals. It is possible that in the SED group, the decrease in % BF was due to a higher initial value as a minimal increase in % BF occurs in individuals who are overweight. On the other hand, the lower overall % BF one has, the more difficult it is to lose BF. These results are similar to those supporting a maintenance of % BF with age, especially over age 60[34,35] or a decrease in % BF in late old age[36]. Furthermore, these results are also in accord with studies showing no change or minimal change in BW after early old age (over age 50)[37]. Another possibility is that perhaps this group of sedentary individuals may have made dietary modifications, which largely influenced their % BF. However, dietary data collected in questionnaires were insufficient for meaningful analysis.

Hypertension is well recognized as the most potent, common, and determinable risk factor for cardiovascular disease in elderly individuals, whereas physical activity is widely recommended as a useful adjunct in primary and secondary prevention of hypertension in sedentary elderly individuals. In the HA group under study, results suggested that maintaining highly active lifestyles helped to preserve their initial (i.e., before the exercise training) resting and maximal BP. However, for the MOD active elderly individuals, the results indicated a slight but significantly increased SBPmax (5%), maximal DBP (DBPmax) (8%), and RDBP (8%) over the follow-up period. These observations differ from the findings of a study showing 6 months of continuous endurance walking exercise training eliciting a beneficial effect in reducing RBP in elderly normotensive subjects[18]. Studies have shown that elderly men and women with lower fitness levels have significantly higher average resting arterial BP than those in excellent fitness categories[38]. In support of these studies, the present study indicated that the SED group averaged 6% elevation in RSBP and RDBP for the longitudinal analysis (T1 to T3 analysis). Furthermore, our data indicated that the SED elderly individuals were able to somewhat maintain their RBP values similar to values recorded before training (T1). These data suggested that the nature of activity undertaken during the follow-up period was probably different from the one attained throughout the training studies. Thus, activity should be continued in order to maintain beneficial effects of exercise. It is important to mention that the RBP, although slightly elevated, was continually far below 160/95 mm Hg, which is less than the RDBP value considered as a risk factor for CHD, therefore, reflecting reduced risk of CHD for all groups. Considering their lack of activity, it is possible that the SED group was able to maintain average normal RBP due to preferable genetic factors, possible modification of nutrition, and maintenance of % BF and BW. In addition, one of the limitations of this study was that a normal RBP was initially necessary in order for the person to participate in the present study. Therefore, conclusions with regard to RBP and lifestyle status are limited.

Advanced age is associated with a decline in FEV/FVC for almost every subgroup with the rate of FEV1.0 decline accelerating in advancing years. The present longitudinal analysis showed a slight decrement (~10%) in pulmonary function for the HA and MOD elderly individuals in agreement with the literature[35]. As we anticipated, data indicated that sedentary individuals experienced even greater decreases in pulmonary function over time. In contrast, results for the SED group also indicated higher FEV/FVC values. Therefore, it would seem reasonable to speculate that these higher FEV/FVC values may be an indication for developing restrictive lung disease in these sedentary individuals. However, it is difficult to reach any clear conclusions regarding pulmonary measurements owing to the limited number of observations for the pulmonary variables. It is important to keep in mind that the same number of subjects for the HA and MOD groups yielded different results.

One of our main purposes in the present study was to evaluate current physiological status between the different study categories, since many studies support the finding that elderly individuals who maintain an active lifestyle have a healthier physiological profile and a more functional life than those who are significantly less active[39]. If so, our physically active elderly individuals should have better physiological functioning compared to age-matched sedentary elderly individuals. Indeed, elderly individuals with habitual higher levels of physical activities, had better results on tests of % BF, PPT, and sum of seven skinfolds. However, no differences were found between groups for resting arterial blood pressure, blood lipids, aerobic capacity, and pulmonary function. Overall, $VO_{2max}$, RBP, PPT, and % BF were found to be consistently favorable for the HA and the MOD groups compared to the SED group. Our results are consistent with a study by Voorrips et al.[40], who reported that elderly women with habitual higher physical activities had better endurance, RBP, and peak expiratory flow. Although RSBP and RDBP showed a tendency toward a small, but nonsignificant reduction for the HA group, it was considered unlikely that normotensive elderly subjects such as those in the present study would benefit from active lifestyles related to BP reduction observed in subjects with mild or borderline hypertension[41].

The role of regular physical activity in the etiology of chronic diseases associated with aging has been increasingly recognized over the past few decades. The daily functional capacity of individuals is reduced well before any physiological functional capacity is decreased and disease emerges. The PPT is well accepted as a practical tool for estimating functional capacity through the ability to perform certain activities of daily living[26]. In the present study, sedentary elderly individuals had lower scores on the PPT test compared to the moderately active and highly active subjects. Thus, it appeared that the PPT should be used as a method of choice to estimate functional capacities of individuals.

Finally, an active lifestyle can result in higher levels of HDL and lower levels of LDL[42]. In contrast to other measurements, LDL, HDL, and cholesterol measurements were not influenced by activity levels in our study. It is possible that active lifestyles are very unlikely to improve plasma lipid profiles unless the activities are accompanied by substantial weight loss or changes in dietary habits.

One limitation of this study is the lack of quantifiable information about the activity status of the subjects during the period from the termination of the initial studies to the present investigation. It is certainly possible that a number of subjects had changed their activity levels during the 3- to 7-year interval from the initial to the present study.

## CONCLUSIONS

In summary, the present study could not clearly demonstrate favorable differences for the active groups over the sedentary group with regard to several important physiological factors over the 5-year follow-up and it appears that the recommendation for, and the initiation of, adopting active lifestyles may not be sufficient on their own to significantly increase an individual's physiological functioning.

A striking and somehow unexpected finding of the present study was the demonstration that many important physiological parameters do not differ significantly between sedentary and active elderly individuals. Furthermore, given the preferable results for the active group on most tested variables, we suggest that misclassification is unlikely to have biased our results. Perhaps even more important, our data might suggest that the average elderly person does not participate in sufficiently high intensity exercise levels to provide most of the disease prevention benefits associated with increased physical activity levels shown in many training studies. Although the health benefits of regular physical activity regarding a variety of chronic disease areas, including cardiovascular disease, are now well established, many elderly people do not engage in regular physical activity. In addition, those who exercise do not perform it with appropriate frequency or intensity required to obtain positive physiological changes such as improved cardiovascular fitness. Moreover, these results suggest that unsupervised self-engaged physical activity may not always benefit elderly people.

## ACKNOWLEDGMENTS

This manuscript is dedicated to the memory of professor Michael L. Pollock, Ph.D. (1936–98). Michael L. Pollock was the Director of the University of Florida Center for Exercise Science and suffered an aneurysm while attending the annual meeting of the American College of Sports Medicine (ACSM). He had been responsible for new national exercise guidelines, updated every 10–12 years, based on committee reviews of published scientific papers. Pollock chaired the ACSM sponsored group since its inception in 1972. His career research highlights includes a 20-year study of master athletes 40 years and older that yielded information on the effects of aging on aerobic capacity, developing "exercise prescriptions" that hasten recovery after heart surgery, demonstrating that proper exercise programs can increase aerobic capacity 15–30% in people age 30 and older, determining low-impact exercises such as rapid walking to be better than jogging for the elderly, showing that building stronger bones through weight training can help prevent osteoporosis, and determining that one set of weight-lifting exercises may be just as beneficial as the three-consecutive-set regime commonly performed. Pollock was the recipient of several awards, including a Fellowship Award for Research from the Japan Society for the Promotion of Science, an Award of Excellence from the American Association of Cardiovascular and Pulmonary, and the 1993 Scholar Award from the American College of Sports Medicine, Southeastern Region. He authored more than 275 articles, 3 books, and 2 monographs on exercise physiology, physical fitness, cardiac rehabilitation, and sports medicine. He was past president of the ACSM and a fellow of both the American College of Cardiology and the American

Association of Cardiovascular and Pulmonary Rehabilitation. From 1972–1989, he served as a consultant to the President's Council on Physical Fitness and Sports.

## BIOSKETCHES

**Eli Carmeli, P.T., Ph.D.,** is currently working in a full-time tenure tract position at the Physical Therapy Department, Sackler Faculty of Medicne, Tel Aviv University. He is investigating the aging process both on the cellular and clinical level. Email: elie@post.tau.ac.il

**Pini Orbach, Ph.D.,** Cardiovascular Research Center, Massachusetts General Hospital, Harvard Medical School, Boston, MA. E mail: porbach@minervabio.com

**David T. Lowenthal**, **M.D., Ph.D**., is a Professor of Medicine, Pharmacology, and Exercise Science at the University of Florida, Gainesville. He came to the University of Florida in 1988 to serve as Director of the Geriatric Research, Education and Clinical Center (GRECC). Research interests involve pharmacologic responses in aging and exercise, exercise responses in the healthy and frail elderly, hypertension and vascular dementia, and the treatment of the elderly hypertensive. He is a member of 13 editorial boards including *Clinical Nephrology*, *Journal of Geriatric Nephrology and Urology*, *Clinical Pharmacology & Therapeutics*, and *Journal of the American Geriatric Society*. He has been funded by the American Heart Association, the American Lung Association, the American Federation for Aging Research, and numerous grants for clinical trials from the pharmaceutical industry. E-mail: dlowen4241@bellsouth.net. Website: www.med.ufl.edu/pharm/facdata/GRECC/cvlowen.html

**Joav Merrick, M.D., D.M.Sc**., is Professor of Child Health and Human Development affiliated with the Zusman Child Development Center and Division of Community Health at the Ben Gurion University, Beer-Sheva, Israel and presently the Medical Director of the Division for Mental Retardation, Ministry of Social Affairs, Jerusalem and the Director of the National Institute of Child Health and Human Development. He has numerous publications in the field of child and human development, rehabilitation, intellectual disability, disability, health, welfare, abuse, advocacy, and prevention. Dr. Merrick received the Peter Sabroe Child Award for outstanding work on behalf of Danish Children in 1985 and the International LEGO-Prize ("The Children's Nobel Prize") for an extraordinary contribution towards improvement in child welfare and well being in 1987. E-mail: jmerrick@internet-zahav.net. Website: www.nichd-israel.com

**Raymond Coleman,** is Professor at the Department of Anatomy and Cell Biology, Bruce Rappaport Faculty of Medicine, Technion-Israel Institute of Technology, Haifa, Israel. E-mail: coleman@tx.technion.ac.il
